# Characteristics of role models who influenced medical residents to choose surgery as a specialty: exploratory study

**DOI:** 10.1590/1516-3180.2017.0053030517

**Published:** 2017-11-06

**Authors:** Carlos Eli Piccinato, Maria de Lourdes Veronese Rodrigues, Laura de Andrade Rocha, Luiz Ernesto de Almeida Troncon

**Affiliations:** I MD, PhD. Full Professor, Vascular and Endovascular Surgery Division, Department of Surgery and Anatomy, Faculdade de Medicina de Ribeirão Preto (FMRP), Universidade de São Paulo (USP), Ribeirão Preto (SP), Brazil.; II MD, PhD. Full Professor, Ophthalmology Division, Department of Ophthalmology, Otorhinolaryngology and Head and Neck Surgery, Faculdade de Medicina de Ribeirão Preto (FMRP), Universidade de São Paulo (USP), Ribeirão Preto (SP), Brazil.; III MD. Postgraduate Student, General Surgery Program, Faculdade de Medicina de Ribeirão Preto (FMRP), Universidade de São Paulo (USP), Ribeirão Preto (SP), Brazil.; IV MD, PhD. Full Professor, Gastroenterology Division, Department of Internal Medicine, Faculdade de Medicina de Ribeirão Preto (FMRP), Universidade de São Paulo (USP), Ribeirão Preto (SP), Brazil.

**Keywords:** Education, medical, Internship and residency, Career choice, Faculty, General surgery

## Abstract

**CONTEXT AND OBJECTIVE:**

Choosing a medical specialty and making decisions concerning a career are difficult processes for medical students and newly graduated physicians. This exploratory study aimed to investigate the influence of role models on the choice of surgery as a career, and to determine the most influential model characteristics.

**DESIGN AND SETTING:**

Qualitative analysis on responses to a self-administered questionnaire, in different teaching-learning settings.

**METHODS:**

Residents from all years of various surgical subspecialties in a university hospital were included in a survey about the factors that determined their choice of surgery. The questions included items on whether a role model had influenced them in choosing surgery, and the personal or professional characteristics of the models that had been most influential. The responses were subjected to qualitative content analysis.

**RESULTS:**

Sixty-four out of 96 medical residents participated. Fifty-three residents (82.8%) acknowledged the influence of role models. Sixteen model characteristics were indicated as important, with 136 mentions. Characteristics classified as technical skills (55%), such as “medical knowledge” and “manual dexterity” predominated over humanistic characteristics (35%), such as “patient-physician relationships” and “ethical behavior”. However, this difference was not statistically significant (Fisher test, P = 0.11). There were no age differences regarding the proportions mentioning “technical” and “non-technical” attributes, but female residents mentioned significantly more technical skills than their male colleagues did.

**CONCLUSIONS:**

The influence of role models seems to be an important factor determining the choice of surgery as a career. The influential characteristics of the models include not only technical but also humanistic qualities.

## INTRODUCTION

Choosing a medical specialty and making decisions concerning a future career comprise complex and difficult processes for medical students and newly graduated physicians. These processes are important not only for individuals but also for healthcare systems, as they have implications in medical workforce planning.^1^^,^^2^

A number of different factors are known to influence medical specialty choice, including gender, personality features, academic interests, specialty characteristics and training demands, curricular and extracurricular past experiences, financial aspects and lifestyle expectations.^1^^,^^2^^,^^3^ Among these influential factors, role models, such as teachers, tutors and supervisors, to whom medical students and trainees are exposed, embody one of the most important influences on medical specialty choice.^4^

Surgical specialties may be characterized as being physically and mentally demanding careers, with long and hard training periods, and with diminished potential for lifestyle control.^5^ Regarding the factors influencing the choice of surgery as a career, an investigation carried out in the United Kingdom among medical students found that, for those interested in surgical specialties, a number of perceived career characteristics, such as completing a clinical attachment in this area, professional prestige, technical challenge and prospects of financial rewards were important factors.^3^ An extensive review of published work in Australia found that positive past experiences in a surgical environment, perceived prestige and expectations of higher financial reward were significantly associated with choosing a surgical specialty as a career.^6^

A number of studies have shown that, besides the previously mentioned factors, medical students interested in pursuing surgery as a career, as well as trainees who have already chosen this professional field, have acknowledged the influence of mentors and role models.^7^^,^^8^^,^^9^^,^^10^^,^^11^ A few studies have attempted to identify characteristics and traits associated with successful mentors and role models.^8^^,^^9^^,^^10^^,^^11^^,^^12^ Nevertheless, the influence of role models upon the choice of surgery as a career among newly graduated physicians and, in particular, the attributes and characteristics of these models that are more effective in influencing their choice towards a surgical field have not been much investigated.

## OBJECTIVE

Therefore, the aim of this exploratory study was to investigate the influence of role models among medical residents who had already chosen surgical specialties, and to identify the most influential characteristics of their role models.

## METHODS

### Participants and ethics

The study included medical residents from all years (1^st^ to 5^th^) of the 10 surgical subspecialty training programs of the Department of Surgery of the University Hospital of the Ribeirão Preto Medical School (Faculdade de Medicina de Ribeirão Preto, FMRP). All medical residents were contacted and only those who refused to participate or were away from the Department for electives or congresses were not included.

This was done in accordance with a study project that had been approved by the local Institutional Ethics Committee (Pr. 411/2017). Residents were included after being fully informed about the study aims and were given guarantees regarding anonymity and confidentiality issues.

### Study development

The study was carried out during the first six weeks of the 2014 academic year. Residents were approached individually by one of authors (CEP or LER), while in different practice and learning settings used by the various residency programs. After consent had been obtained, a questionnaire form was handed to the participant, who was invited to record his/her answers immediately and anonymously. The participants were instructed to ask the researcher for clarifications if necessary, but none of them needed to do this. The median time spent in answering the questionnaire was 10 min.

### Instrument

The questionnaire contained items asking for demographic data and only two single open-ended questions addressing the main objectives of the study:

1. “Do you consider that a surgeon acting as a role model due to his/her knowledge, skills, attitudes and values has influenced you in your medical specialty choice?”
2. If your answer to the previous question was positive: “which were the most influential personal or professional characteristics of your role models?”.

### Data analysis

Answers to the first question were recorded as “yes” or “no” and expressed as the corresponding percentages. Answers to the second open question were subjected to content analysis using a standard qualitative technique^13^ for capturing both the manifest (explicit) and the latent (implicit) meaning of every individual response. The responses were read by two of the authors (CEP and MLVR), who jointly coded each of them into different categories, which were expressed as single keywords or expressions. Coding was based on well-known attributes of role models who influence medical specialty choice.^8^^,^^9^^,^^10^^,^^11^^,^^12^ Subsequently, the senior author (LEAT) empirically classified the characteristics mentioned into three groups:

1. “Technical skills”;
2. “Humanistic characteristics”; and
3. “Miscellaneous”.

For the statistical analysis, the latter two classes were aggregated into a category of “non-technical attributes”.

### Statistical analysis

Differences between the proportions of the characteristics of role models pertaining to the classes of “technical skills” and “non-technical attributes”, for the whole sample of participants, as well as between male and female and between “older” and “younger” residents, were analyzed using the nonparametric Fisher exact probability test. For the analysis concerning age, the cutoff point was the participants’ median age (“younger” ≤ 28 years; “older” > 28 years). Differences associated with P-values of less than 0.05 were regarded as statistically significant.

## RESULTS

Sixty-four out of the 96 current residents (66.6%) were surveyed in this study. Participants from three programs (General Surgery, Vascular Surgery and Upper Digestive Tract Surgery) comprised almost two-thirds of the study sample, whereas the remaining subspecialties (Urology, Neurosurgery, Colo-Proctology, Plastic and Reconstructive Surgery, Pediatric Surgery, Cardiovascular Surgery and Head & Neck Surgery) were represented by at least one resident (median 3; range: 1-8). There were 49 male residents (74.2%) and 17 female residents (25.8%). The median age was 28 years. Forty-nine participants (74.2%) had already completed a two-year training period in General Surgery either in our institution or elsewhere, before entering a particular local surgical specialty training program.

Fifty-three residents (82.8%) acknowledged that role models influenced them in their medical specialty choice, while eight had a negative answer and three were not sure about this influence. The 53 medical residents acknowledging the influence of role models mentioned 136 remarkable characteristics overall, which were coded into 16 categories. These results are shown in Table 1.

**Table 1.**
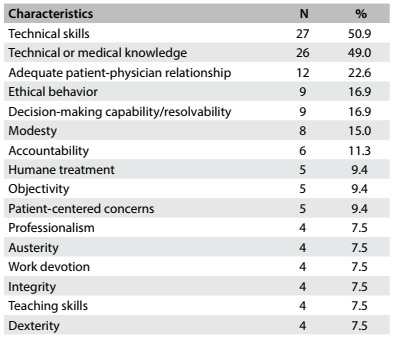
Remarkable characteristics of role models influencing medical residents who chose a surgical specialty as a career. Data are presented as the number (N) and percentage (%) of the 53 residents mentioning each characteristic

These 16 categories were subsequently grouped into three main classes. The “technical skills” class (52.2%) comprised the following categories: technical skills, technical or medical knowledge, decision-making capability/resolvability, objectivity and dexterity. The class of “humanistic” characteristics (31.6%) included the following categories: adequate patient-physician relationship, ethical behavior, modesty, humane treatment, patient-centered concerns and integrity. The class of “miscellaneous” characteristics (16.1%) consisted of the following characteristics: accountability, professionalism, teaching skills, work devotion and austerity.

Although the characteristics classified as “technical skills” were more frequently mentioned than the “non-technical attributes” (52.2% versus 47.7%), this difference did not reach statistical significance (P = 0.11).

There were no significant differences (P = 0.50) between “younger” and “older” participants regarding the proportions of mentions of “technical skills” and “non-technical attributes”. However, female residents, who formed nearly one quarter of the sample of participants, accounted for 70.5% of the mentions of “technical skills” (P = 0.03).

## DISCUSSION

The results from this exploratory study confirm that a large proportion of medical residents in training in several fields of surgery do acknowledge being influenced by a role model, and that technical skills were the most influential model characteristic. Additionally, although humanistic qualities were mentioned by a smaller proportion of the participants than the proportion who indicated that technical skills were most influential, they were cited by more than one third of the participants. Altogether, “non-technical” characteristics were cited nearly as frequently as technical qualities (61/136 versus 75/136), as the most prominent feature of role models who exerted an influence on the respondent’s choice of surgery as a career.

The finding that the majority of the participants in this study recalled being influenced by a surgeon who acted as a role model is in line with several other published studies.^3^^,^^4^^,^^7^^,^^9^^,^^10^^,^^14^ It is plausible, however, that the fact that our instrument only had two single questions may have biased these results. Indeed, the first direct question on whether a surgeon acting as a role model had influenced the participant’s specialty choice could have elicited a positive response bias. However, the percentage of respondents answering positively was comparable to those reported in similar studies using different methods.^3^^,^^4^^,^^9^ Furthermore, the second question, relating to the characteristics of the influential role model is likely to have elicited further thoughts on the topic, and therefore to have had a confirmatory effect regarding the first answer.

The most influential characteristics of role models within surgery, as spontaneously mentioned by the participants of our study, were comparable to those reported by Healy et al.,^10^ who used a structured questionnaire to survey both medical students and surgical trainees. These characteristics were grouped into four categories: clinical competence, personal qualities, teaching abilities and research abilities.^10^ It is noteworthy that many influential characteristics mentioned in the present study were very similar to those reported by Healy et al.,^10^ such as “good surgical technique”, “excellent clinical knowledge” and “compassionate and caring”. However, the meaning of some characteristics mentioned by participants in the present study, such as “professionalism”, “modesty” and “austerity” would deserve clarification through content analysis on data from personal interviews with the respondents. This was beyond the scope of this exploratory study, but may form the focus in further investigations. Nevertheless, it seems that the medical residents in our study were more concerned with non-technical skills than were the surgical trainees included in the study by Healy et al.,^10^ even though they were comparable with the medical students included in their study. This difference can possibly be explained by cultural differences between the respective groups of participants in these studies.

It is well known that the influence of role models is one of the main factors determining medical specialty choice.^3^^,^^4^^,^^15^ This influence appears to be exerted similarly in both surgical and non-surgical specialties and has been reported in studies from different countries^1^^,^^2^^,^^3^^,^^6^^,^^7^^,^^14^^,^^16^ Nevertheless, the findings from a study by Ibrahim et al.^3^ suggest that role models are likely to be more influential for those pursuing a surgical specialty than for those interested in nonsurgical fields. Whereas the latter group appears to be more influenced by factors such as length of training and lifestyle, the former group acknowledged the influence of role models, despite also mentioning factors such as prestige and prospects of financial reward.^3^

Role models have been defined as “individuals admired for their ways of being and acting as professionals*”*.^17^ Teachers, trainers and also senior trainees may have excellent professional performance, together with humanistic qualities, such that they may serve as role models for students and other trainees within healthcare professions. These same qualities may attract people to seek their professional services.^18^^,^^19^^,^^20^

As mentioned in a recent study,^21^ it is important to differentiate role models from mentors, since models exert their influence only by example, whereas mentors have a more formal and lasting relationship with students. It appears that the interaction between students and trainees and their role model depends not only on the model’s characteristics but also on the choices made by the model’s followers.^21^ These influences and relationships need to be taken into consideration in the current changing world of professional education. Indeed, over the last few decades, medical and healthcare professionals’ education and training have undergone a number of changes that have influenced teacher and trainer roles. In a seminal essay published in 2000, Harden and Crosby^22^ described twelve different roles of teachers, which were grouped into six diverse categories according to student or trainee learning scenarios, and included mentors and role models.

Both mentors and role models should be regarded as useful resources when implementing strategies aimed towards recruiting medical students to choose surgical fields as the medical specialties for their careers. These strategies are likely to become increasingly necessary, given that there have been reports of both decreasing numbers of candidates for surgical careers^23^ and increasing attrition rates amongst surgical trainees in some countries.^24^ It has been suggested that, in planning teaching experiences and in actually teaching, factors that are known to negatively influence the choice of surgery as a career should be worked on, as a way of improving recruitment.^25^ Likewise, it has been suggested that medical students should be exposed to positive surgical experiences as early as possible.^9^ Nevertheless, the contribution of surgical mentors and role models and the importance of their actions, starting from the earliest phases of medical education, have been greatly stressed.^10^

While a few teachers and other staff may have natural skills and characteristics that favor them as mentors and role models, it is plausible that the necessary attributes could be acquired though systematic learning in teacher development programs, as suggested elsewhere.^22^ In this regard, the results from our study suggest that professionals acting as role models are more likely to positively influence potential candidates to a career in a surgical field when exhibiting not only good technical skills and other well-known attributes of good surgeons (such as strongly grounded medical knowledge and decision-making capability), but also more humanistic qualities, including an adequate patient-physician relationship, ethical behavior, modesty, humane treatment and patient-centered concerns. These findings should therefore be taken into account by academic departments of surgery in recruiting teachers and devising teacher development programs.

Furthermore, acquisition of these characteristics should be regarded as a goal within general surgical education and training, since both senior surgical trainees and practicing surgeons may act as role models to influence medical students and junior trainees to pursue a career in a surgical field. Firstly, they should be aware of their potential influence, which has been reported as being underestimated.^26^ Additionally, as much as possible, they should be educated to acquire not only the necessary professional skills but also humanistic qualities.

The finding that female residents mentioned technical skills more frequently than did their male colleagues was rather surprising, since among medical students women have tended to show more positive attitudes than men towards humanistic factors involved in healthcare work, such as empathy^27^ and patient-centered care.^28^ Although this finding deserves confirmation through further studies, it is tempting to speculate whether this trait is particular to female physicians choosing surgery as a career.

This study has two main limitations: the data came from a single training center and the participants comprised a relatively small sample. Nevertheless, many of these residents had completed previous training periods in General Surgery at other institutions throughout the country and the array of characteristics mentioned as having influence were rather diversified. Moreover, notwithstanding the exploratory nature of the present study, its main results and particularly those relating to the influence of a role model on the choice of medical specialty were in line with published data originating from many different centers and countries.

Other study limitations that must be acknowledged relate to the survey instrument and the study sample. Concerning the questionnaire, no formal validation procedures were applied, and this can be justified by the exploratory nature of this study. Additionally, the first open question, as mentioned earlier and already discussed, could have elicited a positive response bias. Regarding the study sample, it only included surgical residents expressing their retrospective perceptions of the influence of role models on their choices. Further studies also including residents in non-surgical fields such as Internal Medicine and Pediatrics, with the aim of ascertaining reasons for not choosing surgery, may expand the knowledge of factors affecting medical specialty choices, thus revealing characteristics of role models that could favor or even hinder the choice of surgery in particular as a career.

## CONCLUSIONS

As in other medical specialties, the influence of surgeons acting as role models comprises an important factor in determining the choice of surgery as a career. The role model characteristics that are regarded as being most influential include not only technical skills, which are intrinsically linked to surgical practice, but also humanistic qualities, such as adequate patient-physician relationship, ethical behavior, modesty, humane treatment and patient-centered concerns.

## References

[1] Querido SJ, Vergouw D, Wigersma L (2016). Dynamics of career choice among students in undergraduate medical courses. A BEME systematic review: BEME Guide No. 33. Med Teach.

[2] Correira Lima de Souza L, Mendonça VR, Garcia GB, Brandão EC, Barral-Netto M (2015). Medical Specialty Choice and Related Factors of Brazilian Medical Students and Recent Doctors. PLoS ONE.

[3] Ibrahim M, Fanshawe A, Patel V (2014). What factors influence British medical students’ career intentions?. Med Teach.

[4] Passi V, Johnson N (2016). The impact of positive doctor role modeling. Med Teach.

[5] Bland KI, Isaacs G (2002). Contemporary trends in student selection of medical specialties: the potential impact on general surgery. Arch Surg.

[6] Grigg M, Arora M, Diwan AD (2014). Australian medical students and their choice of surgery as a career: a review. ANZ J Surg.

[7] Erzurum VZ, Obermeyer RJ, Fecher A (2000). What influences medical students’ choice of surgical careers. Surgery.

[8] Nguyen SQ, Divino CM (2007). Surgical residents as medical student mentors. Am J Surg.

[9] Ravindra P, Fitzgerald JE (2011). Defining surgical role models and their influence on career choice. World J Surg.

[10] Healy NA, Glynn RW, Malone C, Cantillon P, Kerin MJ (2012). Surgical mentors and role models: prevalence, importance and associated traits. J Surg Educ.

[11] Healy NA, Cantillon P, Malone C, Kerin MJ (2012). Role models and mentors in surgery. Am J Surg.

[12] Cochran A, Paukert JL, Scales EM, Neumayer LA (2004). How medical students define surgical mentors. Am J Surg.

[13] Campos CJG, Turato ER (2009). Análise de conteúdo em pesquisas que utilizam metodologia clínico-qualitativa: aplicação e perspectivas: [revisão] [Content analysis in studies using the clinical-qualitative method: application and perspectives: [review]]. Rev Latinoam Enferm.

[14] Lawal TA, Afolabi AO (2013). Factors influencing the choice of surgery as a career by pre-registration interns. Afr Health Sci.

[15] Passi V, Johnson S, Peile E (2013). Doctor role modelling in medical education: BEME Guide No. 27. Med Teach.

[16] Sobral DT (2006). Influences on choice of surgery as a career: a study of consecutive cohorts in a medical school. Med Educ.

[17] Irby DM (1986). Clinical teaching and the clinical teacher. J Med Educ.

[18] Frezza EE, Watchel MS (2007). The quality of a surgeon defined by internal medicine and family practice physicians: a closed-ended survey with importance scale. Am Surg.

[19] Piccinato CE, Rodrigues MLV, Rocha L, Troncon LEA (2014). Characteristics of Role Models Who Influenced Medical Residents to Choose Surgery as a Specialty. AMEE.

[20] Musunuru S, Lewis B, Rikkers LF, Chen H (2007). Effective surgical residents strongly influence medical students to pursue surgical careers. J Am Coll Surg.

[21] Passi V, Johnson N (2016). The hidden process of positive doctor role modelling. Med Teach.

[22] Harden RM, Crosby J (2000). AMEE Guide No 20: The good teacher is more than a lecturer - the twelve roles of the teacher. Medical Teacher.

[23] Neuhaus P (2007). Why should young doctors choose to become surgeons?. Ann Surg.

[24] Yeo H, Bucholz E, Ann Sosa J (2010). A national study of attrition in general surgery training: which residents leave and where do they go?. Ann Surg.

[25] Glyn RW, Kerin MJ (2010). Factors influencing medical students and junior doctors in choosing a career in surgery. Surgeon.

[26] Quillin RC, Pritts TA, Davis BR (2012). Surgeons underestimate their influence on medical students entering surgery. J Surg Res.

[27] Santos MA, Grosseman S, Morelli TC, Giuliano IC, Erdmann TR (2016). Empathy differences by gender and specialty preference in medical students: a study in Brazil. Int J Med Educ.

[28] Hardeman RR, Burgess D, Phelan S (2015). Medical student socio-demographic characteristics and attitudes toward patient centered care: do race, socioeconomic status and gender matter? A report from the Medical Student CHANGES study. Patient Educ Couns.

